# Novel Composite Planks Made of Shape Memory Polyurethane Foaming Material with Two-Step Foaming Process

**DOI:** 10.3390/polym14020275

**Published:** 2022-01-11

**Authors:** Jan-Yi Lin, Mei-Chen Lin, Bing-Chiuan Shiu, Ching-Wen Lou, Jia-Horng Lin, Yueh-Sheng Chen

**Affiliations:** 1 Graduate Institute of Biomedical Sciences, China Medical University, Taichung 404333, Taiwan; janyiisme@gmail.com (J.-Y.L.); ritalin2870@gmail.com (M.-C.L.); 2 College of Material and Chemical Engineering, Minjiang University, Fuzhou 350108, Fujian, China; bcshiu@mju.edu.cn; 3 Fujian Key Laboratory of Novel Functional Fibers and Materials, Minjiang University, Fuzhou 350108, Fujian, China; 4 Department of Bioinformatics and Medical Engineering, Asia University, Taichung 413305, Taiwan; 5 Department of Medical Research, China Medical University Hospital, China Medical University, Taichung 404332, Taiwan; 6 Advanced Medical Care and Protection Technology Research Center, College of Textile and Clothing, Qingdao University, Qingdao 266071, China; 7 Advanced Medical Care and Protection Technology Research Center, Department of Fiber and Composite Materials, Feng Chia University, Taichung 407802, Taiwan; 8 School of Chinese Medicine, China Medical University, Taichung 404333, Taiwan

**Keywords:** shape memory polyurethane (SMP), electromagnetic wave shielding, buffer absorption, carbon fibers, sandwich-structured composite planks

## Abstract

In this study, shape memory polyurethane (SMP) foaming material is used as the main material that is incorporated with carbon fiber woven fabrics via two-step foaming method, forming sandwich-structured composite planks. The process is simple and efficient and facilitates any composition as required. The emphasis of this study is protection performances, involving puncture resistance, buffer absorption, and electromagnetic wave shielding effectiveness. The proposed soft PU foam composite planks consist of the top and bottom PU foam layers and an interlayer of carbon fiber woven fabric. Meanwhile, PU foam is incorporated with carbon staple fibers and an aluminized PET film for reinforcement requirements and electromagnetic wave shielding effectiveness, respectively. Based on the test results, the two-step foaming process can provide the PU foam composite planks with excellent buffer absorption, puncture resistance, and electromagnetic wave shielding effectiveness; therefore, the proposed composite planks contribute a novel structure composition to SMP, enabling it to be used as a protective composite. In addition, the composites contain conductive material and thus exhibit a greater diversity of functions.

## 1. Introduction

Owing to the development of technology, shape memory polymer is used as a smart material, demonstrating great application potential in major areas [1,2,3]. In comparison with shape memory ceramics or shape memory alloys, shape memory polymer has more merits, e.g., light weight, a variety of multiple stimulation methods, highly adjustable properties, and easy processing, the merits of which are the emphases of studies on smart polymer materials [2,4,5]. Subsequently, shape memory polymers are commonly used in the biomedical material, self-healing material, smart textile, drug-controlled release, and aerospace/aviation fields [6,7,8,9,10,11]. The common polymer industry usually proceeds production exclusively based on the physical and chemical requirements by the application ends, yet it requires more than basic physical properties when producing shape memory polymer. Shape memory polymer is dependent on the design required by the products otherwise, because the interaction between shape memory polymer and product is highly correlated with the production [12,13,14,15]. As shape memory polymer has a high material cost, many foreign manufacturers use it to produce protection products with a high added value [16,17,18,19,20].

Foaming is frequently utilized in the production of shape memory polyurethane (SMP). Hu et al. established foam models with 76 measures. A single-axis compression was used to simulate the elasticity and plasticity behaviors of close-cell foaming materials in the initial loading stage. The results indicated that the foaming models with a regular cell structure exhibited a greater compression resistance, while the presence of filler was pertinent to the final properties of the products [21]. Li et al. related the cell structure on the electromagnetic wave shielding performance to the SiC/C foaming structure. The cell structure was substantiated to be effective in electromagnetic wave shielding because of diffraction, multiple reflection, and absorption of electromagnetic waves, as well as the improvement in interfacial polarization [22]. Zhu et al. examined the correlation between the low-speed impact resistance and the composites containing multiple foam layers. They developed a multi-layered analysis model that was based on the energy level, thereby predicting the contact force, the displacement of the impactor, and the energy absorption capacity. The test results indicated that, made with the same mass, the multi-layered composites exhibited greater impact resistance than single-layered composites when being exerted with a low impact force [23].

According to the literature, it is found that foaming material has widespread applications, whereas its mono-composition limits the diversity of performances. As a result, it is an inevitable trend to synthesize different types of materials to address the downside of mono-composition foaming materials. In this study, the aim was to produce composites with an efficient, convenient, and feasible soft PU foaming method. By means of multiple foaming processes, PU foam is combined with reinforcing fabrics, forming sandwich-structured PU foam composite planks. The manufacturing process is simple, efficient, and fruitful, and allows the incorporation of carbon staple fibers and carbon fiber woven fabrics without jeopardizing the PU foam’s ability to take shape; therefore, it provides the composite planks with better impact resistance and durability than single-layered PU foam planks at the same mass. PU foam composites can be more mechanically robust via the incorporation of a diversity of fillers or gain more functions via manufacturing processes. Chithra et al. produced PU foam composites that featured thermal insulation and combustion resistance. Natural cotton fibers were dispersed in sucrose water solution, and processed with compression, filtration, drying, and carbonization until reaching consolidation, forming PU foam composites. The PU foam composites had a maximal EMI of −38.9 dB and diverse functions, but they required a complex process [24]. Additionally, Duan et al. produced PU foam composites with epoxy, nickel-coated carbon fibers, carbon nanotubes, and carbon black. The electromagnetic shielding performance of PU foam composites proved that the presence of filler contributed a synergistic effect, building a conductive network inside the PU foam composites. The electromagnetic shielding performance was thus improved to −40.8 dB [25].

Up to date, PU foam that is mechanically robust is commonly incorporated with conductive fillers to attain more functions for a diversity of application based on previous studies [26,27,28]. Moreover, PU foam can be supplied with electromagnetic wave shielding effectiveness (EMSE) via different methods and the shield mechanisms include reflection loss, absorption loss, and multi-reflection loss. Hence, the addition of conductive fillers for EMSE reinforcement employs the absorption loss mechanism exclusively. Absorption loss means the electromagnetic wave energy loss caused by overcurrent when it passes the interior of a shield; therefore, absorption loss is dependent on the thickness, magnetic permeability, and conductivity of materials. Although the presence of fillers can strengthen the EMSE of PU foam composites, it may as well jeopardize PU foam otherwise. For example, the incorporation of fillers may hinder the growth of cells, and an uneven distribution of fillers also leads to a lower evenness of, or even ruptures in cells, which in turn adversely affects the mechanical properties of PU foam composites. As a result, it is essential to investigate and explore the correlation between the content of fillers as well as the functions and the mechanical properties of PU foam.

In addition to the incorporation of ordinary fillers, in this study, common methods are improved with the two-step foaming method being employed. Carbon fiber woven fabrics are thus added in the interlayer during the process, and the interlaced warp and weft carbon fibers build an intact connective network for the composite planks. Meanwhile, aluminum-coated films, that are used as the surface layer, strengthen the composite planks in terms of the reflection loss mechanism of EMSE. Besides, the conduction of the two-step foaming method can combine various materials, providing the composite planks with a stabilized structure and a flexible thickness. The manufacturing process is eco-friendly and is more efficient than other single-layer foaming method. To sum up, the proposed composite planks take the advantage of flexible PU foam structure, and the incorporation of soft textiles keeps the resulting product soft and compact. In addition, the proposed composite planks are mechanically robust and provide electromagnetic wave shielding, which qualify them for the EMSE use, required by aviation, aerospace, and portable electronic products, especially for use in extreme environments.

## 2. Materials and Methods

### 2.1. Materials

Carbon fiber woven fabrics (TORAYCA™, Taipei, Taiwan) have a yarn fineness of 3000 filaments/strand, a warp density of 12.5 nds/inch, and a weft density of 12.5 picks/inch. High density soft PU foaming agents (APEXLON^®^, two-agent type, KUANG LUNG SHING CORPORATION, Taoyuan, Taiwan) include polyol as agent A and isocynate as agent B. Carbon staple fibers (HTS 40, TORAYCA™, Taipei, Taiwan) have a length of 6.2 mm and a diameter of 7 μm. Aluminum-coated PET films (aluminized films) are purchased from GENERAL HORNG company, Taoyuan, Taiwan.

### 2.2. Preparation of PU Foam Composite Planks

**Figure 1:**
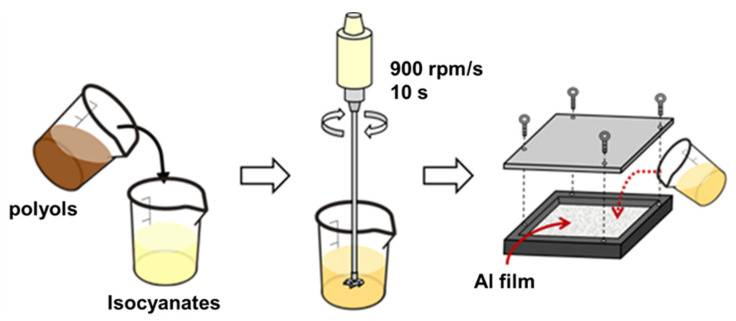
The manufacturing process of PU foam composite planks.

Figure 1 shows the manufacturing process of PU foam composite planks, involving three stages. The planks consist of five layers from top to bottom in the following order: an aluminized film, a PU foam, a carbon fiber woven fabric, a PU foam, and finally an aluminized film. In the 1st stage, polyols and isocyanates are mixed at 900 rpm/min for 10 s, during which 80 g and 120 g of carbon staple fibers (abbreviated as carbon) are separately added, forming different batches. The content of carbon staple fibers accounts for an additional amount of 20% and 30% to the total volume of foaming agent. In our pilot study, we tried to incorporate 40%, 80%, 120%, and 160% of carbon staple fibers with PU foam. It was found that a carbon fiber content lower than 80 g does not provide the composite planks with any electromagnetic shielding effectiveness; whereas, a carbon fiber content exceeding 120 g causes the foaming solution too thick to foam. Hence, the parameter is determined to be 80 g and 120 g of carbon staple fibers in this study.

In the 2nd stage, the PU foaming (abbreviated as PU) mixtures are infused into a mold (320 mm × 330 mm × 20 mm) where an aluminized film (abbreviated as Al) is laid in advance. The mixtures are left still in the sealed mold for a 300 min foaming process, yielding Al film/PU/carbon (hereafter referred to as APC) composite planks. In the 3rd stage, after the APC composite plank is placed in a mold of 320 mm × 330 mm × 40 mm, a carbon fiber nonwoven fabric (abbreviated as W) and then a PU foam mixture are added in order. They are left for 300 min foaming as previously described, yielding APWPA composite planks. The APWPA composite planks are finally evaluated for basic properties. In addition, the samples and composition used in the study are shown in Table 1.

### 2.3. Testing

#### 2.3.1. Dynamic Impact Test

As specified in ASTM D1596, the falling weight impact test is conducted to measure the impact resistance of samples (100 mm × 100 mm) using a semicircular impactor that hits the samples in a manner of free-fall. The energy of impact is 9000 N. Six samples for each specification are used for the test. Based on the record of the server (CHANGFATA Industrial Company, Taichung, Taiwan), the residual impact energy is then computed for impact resistance. Next, the fractured samples collected from the falling weight impact test are severed from the impact site, after which, the cutting section is observed by SEM, thereby evaluating the recovery and mechanical reinforcement theories of PU foam composite planks.

#### 2.3.2. Dynamic Puncture Resistance Test

As specified in NIJ standard 0115.00 (stab resistance of personal body armor), samples (100 mm × 100 mm) are evaluated for puncture resistance (level one protection-E1) with the falling weight stab resistance test using a using a 9000 N semicircular impact tester (CHANGFATA Industrial Company, Taichung, Taiwan). The impact energy is 24 J and the impactor is released from a height of 288 mm. Six samples for each specification are tested for average.

#### 2.3.3. Electromagnetic Wave Shielding Measurement

As specified in ASTM D4935-10 (standard test method for measuring the electromagnetic shielding effectiveness of planar materials), a coaxial transmission clamp and a spectra analyzer (KC901S, TS RF Instruments Co., Ltd., Taoyuan, Taiwan) are used in the electromagnetic wave shielding measurement. Prior to the test, a blank specimen with the same thickness is used as reference, and the electromagnetic shielding effectiveness (SE_ref_) is used to rectify the instrument at a frequency of 100 KHz~3 GHz. Samples have a size of 150 mm × 150 mm and 10 samples for each specification are tested for average.

#### 2.3.4. Vertical Rebound Test

As specified in ASTM D2632-15 (standard test method for rubber property—resilience by vertical rebound), the impactor is released in a free-fall manner from a specified height. A horizontal adjustment is required by the tester (HUNG TA Industrial Company, Taichung, Taiwan) prior to the test, ensuring that the tester is stabilized affixed. The impactor is then lifted to the required height that has a minimal distance away from the sample being 14 mm. The first rebound height is measured, after which the ratio of the rebound height to the impact standard height is computed and recorded as the rebound rate.

## 3. Results and Discussion

### 3.1. Surface Observation of PU Foam Composite Planks

**Figure 2:**
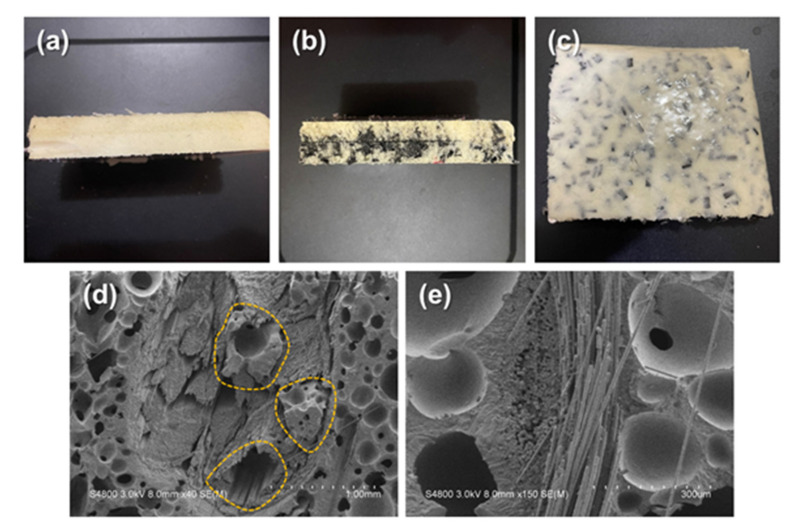
Surface images of (**a**) pure PU foam and (**b**) cutting section, (**c**) the front, and (**d**) interface, and (**e**) magnified interface of composite planks.

Figure 2 shows the image of composite planks where Figure 2a is the pure PU foam with a thickness of 40 mm. Pure PU foam is made by the 2-step foaming process without other additives, while Figure 2b is the PU foam composite planks (i.e., APWPA composite planks, as per Section 2.2). Three different types of materials are well bonded and the resulting composite planks are made with the required shape due to the employment of the PU foaming process. Additionally, Figure 2c shows the outlook of the composite planks where the aluminized film is peeled; meanwhile, carbon staple fibers are well dispersed inside PU foam, indicating that the presence of carbon staple fibers has no negative effects over the constitution of PU foam composite planks. In addition, according to the SEM observation of composite planks, when carbon fiber woven fabrics are combined, foaming could happen in areas like the yellowish zone as Figure 2d. Free foaming has an advantage that within a limited space, foaming material can fill all the space in the zone, the result of which helps mechanically strengthening the composite planks. Figure 2e shows the micro-structure over the interface where fibers are saturated in PU foam and both materials are compounded well. The observation of planks’ surface is supportive evidence that the proposed composite planks made by two-step foaming process acquire a solid morphology in an efficient measure, which in turn suggests that all demands can be satisfied via a diversity of composition according to flexible designs. Hence, different materials can be utilized and combined to suit the applications and occasions.

### 3.2. Buffer Absorption of PU Foam Composite Planks

**Figure 3:**
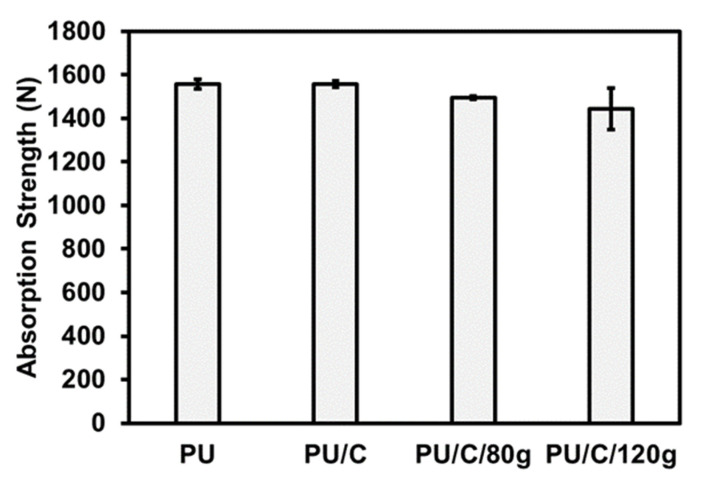
Buffer absorption results of the PU foam composite planks.

In order to buffer absorption, PU foam composite planks are impacted by an impact load of 9000 N for the falling weight impact test. After the sample is damaged to breakage by the impact load, the residual strength is recorded. Figure 3 shows the residual strength of PU foam composite planks as related to the content of carbon staple fibers. Based on the results in Figure 2, regardless of the sample type, the impact load absorption rate is higher than 85%. This phenomenon is attributed to the cells in the foaming materials. When PU foam composite planks are exerted with an external force, cells will be deformed, compressed, and possibly collapsed and destroyed, thereby dissipating the impact energy.

When the content of carbon fiber is increased, the residual strength of the composite PU foam shows a decreasing trend. When there are more carbon fibers incorporated, PU foam is rendered with a limited space in a specified mold volume. Comparing to the control group without fillers, the carbon fiber contained PU foam gains smaller cell size and cells are formed densely. Namely, a higher cell density provides PU foam composites with a more solid supportive structure. Subsequently, when an impact force is loaded, the PU foam composite planks can dissipate a greater impact load while diminishing the residual strength [29,30,31].

**Figure 4:**
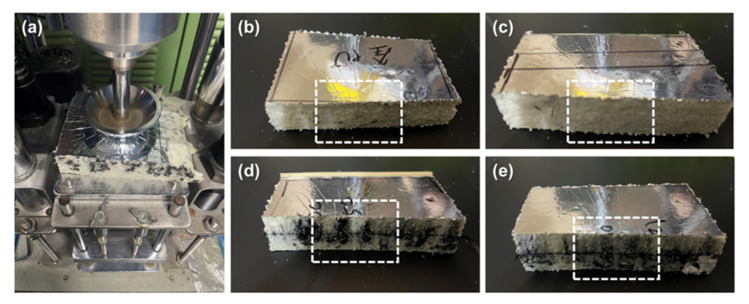
(**a**) Falling weight impact tester, and the corresponding fractured samples of (**b**) PU, (**c**) PU/C, (**d**) PU/C/80 g, and (**e**) PU/C/120 g.

**Figure 5:**
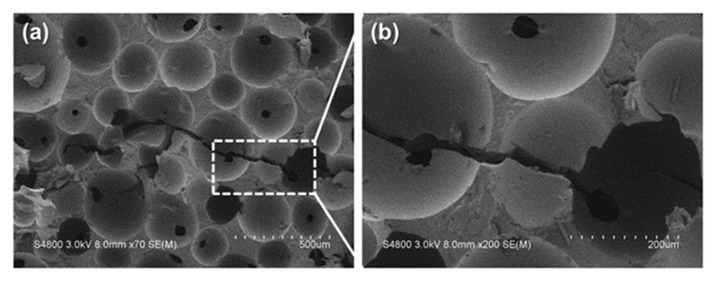
(**a**) SEM image and (**b**) magnified image of fractured site of composite planks after the impact test.

Figure 4 shows the images of fractured sample after the falling weight impact test, as in Figure 4a, where the impactor hits the center of samples in a free-fall manner. As per Figure 4b–e, samples that are rendered with a dent can recover and almost sustain the original state. Because the SMP in the composite planks possesses resilience, the composite planks possess higher durability than metallic or plastic materials and a better cushion effect. Figure 5 shows the SEM images of fractured sites where the cells are ruptured. Although the cells may exhibit cracks, the cracks retain the original structure without exacerbating. The carbon staple fibers provide reinforcement so that the impact force can be effectively stopped by the distribution of staple fibers in the composite planks.

### 3.3. Puncture Resistance of PU Foam Composite Planks

**Figure 6:**
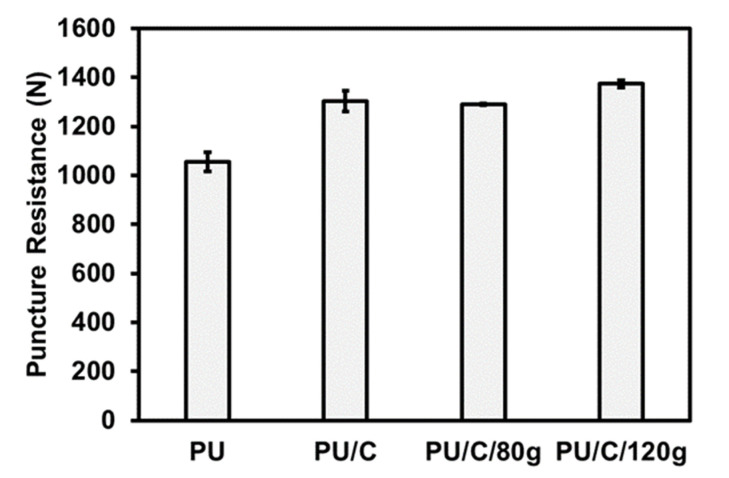
Puncture resistance of PU foam composite planks in the falling weight stab resistance test.

**Figure 7:**
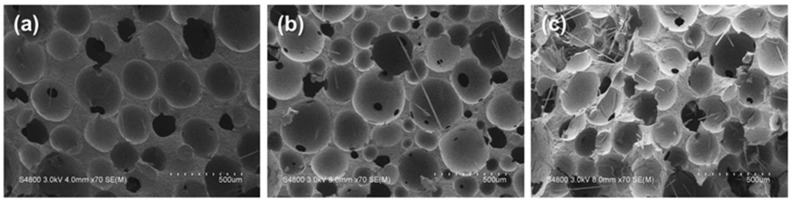
SEM images of (**a**) PU, (**b**) PU/C/80 g, and (**c**) PC/C/120 g.

Figure 6 shows the puncture resistance of the PU foam composite planks in the falling weight puncture resistance test as related to the content of carbon staple fibers. The test results indicate that a rise in the content of carbon staple fibers has a positive influence over the puncture resistance of the PU foam composite planks. The density of the PU foam is increased due to the incorporation of carbon staple fibers as well as a rise in the fiber content, which eventually strengthens the puncture resistance of the PU foam composite planks. In accordance with the observation by the SEM images in Figure 7, compared with the control group (pure PU foam w/o carbon staple fibers), composite planks demonstrate a cell density that is dependent on the content of carbon staple fibers. Moreover, the foaming occurs in a sealed mold, which means, equivalently, that the PU foam has a specified volume. When incorporated with fibers and fabrics, PU foam is deprived with more space during the foaming process [32,33,34]. As a result, the cells over the foaming surface are compressed and constrained from foaming, which in turn causes a denser foamed skin of the PU foam composite planks. The surface of composite planks is firmly formed, demonstrating a greater puncture resistance. Additionally, after the needle-like probe penetrates, the densely formed PU foam composite planks can cause more powerful friction resistance against it. The greater the foaming density, the higher the puncture resistance. Besides, the group containing carbon fiber woven fabric exhibits higher puncture strength, which proves that the two-step foaming structure exhibits greater effect when used in the protection field.

**Figure 8:**
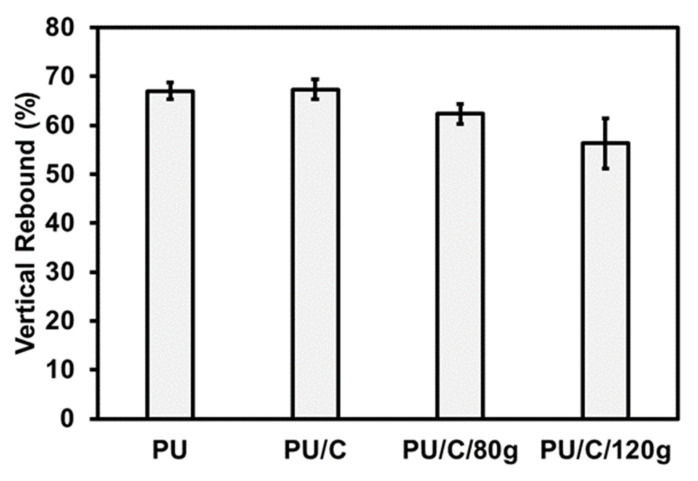
Vertical rebound rate of PU foam composite planks.

Figure 8 shows the vertical rebound rate of PU foam composite planks as related to the content of carbon staple fibers. With the content being increased from 0 g to 120 g, the PU foam composite planks obtain increasingly greater density and a decreasing vertical rebound rate. When there are more carbon staple fibers, there is an increasingly higher foaming density, which hampers the cells of PU foam from foaming because of the compression in a limited space and a saturated condition. As a result, the surface of PU foam composite planks is a lowly foaming skin with a greater surface density, and a rise in the content of carbon staple fibers adversely affects the vertical rebound rate of PU foam composite planks.

### 3.4. Electromagnetic Wave Shielding Effectiveness of PU Foam Composite Planks

**Figure 9:**
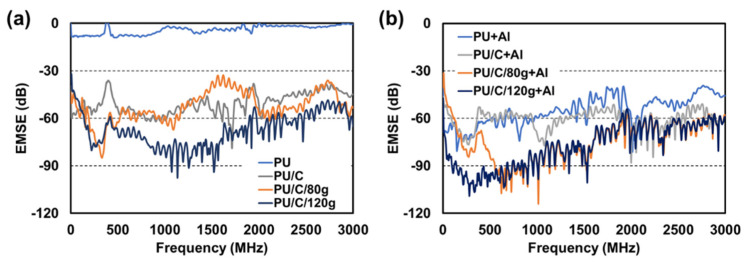
Electromagnetic wave shielding effectiveness of the PU foam composite planks, as related to (**a**) the content of carbon staple fibers and (**b**) the presence of aluminized film.

Figure 9 shows the electromagnetic wave shielding effectiveness of PU foam composite planks. When electromagnetic waves enter a shield, they are absorbed, reflected, and multi-reflected by the surface and interior or penetrate the shield via different loss mechanisms [35,36,37,38,39]. In this case, the electromagnetic energy is consumed by the shield, thereby attaining electromagnetic wave shielding effectiveness. Figure 9a shows the influence of the incorporation of content of carbon fibers over the electromagnetic wave shielding effectiveness of PU foam composite planks. The test results indicate that the pure PU foam (i.e., the control group) fails in providing electromagnetic waves shielding effectiveness. Compared with the control group, when incorporated with more carbon staple fibers, the PU foam composite planks demonstrate stronger shielding effectiveness. In the interior of the PU foam, carbon staple fibers are evenly distributed and in good contact, constructing an effective shielding network. When electromagnetic waves reach carbon staple fibers that have electrical conductivity, the electrons in the PU foam composite planks are prone to activation, and they are able to leap among energy levels, thereby attenuating electromagnetic energy and attaining electromagnetic wave shielding effectiveness.

Figure 9b shows the influence of the incorporation of aluminized film over the electromagnetic wave shielding effectiveness of PU foam composite planks. Regardless of whether carbon staple fibers are incorporated, the combination of aluminized film strengthens the electromagnetic wave shielding of the composite planks, upgrading one level. In particular, in the comparatively less competitive range of 1000~2000 MHz, the presence of the aluminized film strengthens the PU foam composite planks to over −90 dB. Due to the excellent electrical conductivity of aluminized film, the PU foam composite planks can reflect the majority of the electromagnetic waves over the surface, after which the carbon staple fibers in the PU foam, as well as in the carbon fiber woven fabric (the interlayer), jointly multi-reflect the electromagnetic waves, blocking the residual electromagnetic waves. In this case, the electromagnetic wave shielding effectiveness of PU foam composites planks is highly improved.

## 4. Conclusions

In this study, SMP and PU foam are used as the matrix, and the two-step foaming process is utilized to combine carbon fiber woven fabrics and carbon staple fibers with the matrix, forming PU foam composite planks. The study aims to provide PU foam materials with greater protection efficacy and a diversity of application potentials. The test results indicate that the combination of carbon staple fibers significantly stabilizes the structure of composite planks and reinforces the electromagnetic wave shielding effectiveness. Additionally, the PU foam composite planks exhibit excellent buffer absorption, suggesting that the combination design of the composite planks is highly helpful as the buffer absorption exceeds 85%. Moreover, the presence of the interlayer (i.e., the carbon fiber woven fabric) effectively protects the composite planks from being punctured. At last, the results of the electromagnetic wave shielding measurement indicate that the incorporation of the outer layer (aluminized film) achieves an amazing level of over −90 dB. In conclusion, this study proposes a new perspective with a two-step foaming method that synthesizes different materials, the production of which is easy and efficient, and the structure is solid without compromising the performances of the matrix, while adding better mechanical and functional features.

## Figures and Tables

**Table 1:** The composition of the PU foam composite planks.

	Total Number of Layers	Carbon Fiber Woven Fabric	Carbon Staple Fiber	Al Film	Density (kg/m^3^)
PU (Control)	1	−	−	−	−
PU/C	3	−	−	−	156.25
PU/C/80 g	5	+	80 g	+	218.75
PU/C/120 g	5	+	120 g	+	250

## Data Availability

All data relevant to the study are included in the article.
